# Supplementary material for: Resolving the full spectrum of human genome variation using Linked-Reads
Source: Genome Res. 2019 Apr;29(4):635–45. doi: 10.1101/gr.234443.118 (PMC6442396; doi:10.1101/gr.234443.118)
Supplement: Supplemental Material [file supp_gr.234443.118_Supplemental_longranger-2.2.zip › longranger-2.2.tar.bz2!/lr2.2-public/lib/stan/cmdstan/stan/doc/api/html/classstan_1_1common_1_1dump__factory.html]

Stan: stan::common::dump\_factory Class Reference


|  |  |
| --- | --- |
|  | Stan  2.5.0  probability, sampling & optimization |


- Main Page
- Related Pages
- Namespaces
- Classes
- Files

- Class List
- Class Index
- Class Hierarchy
- Class Members

All Classes Namespaces Files Functions Variables Typedefs Enumerations Enumerator Friends Macros Pages

- stan
- common
- dump\_factory

Public Member Functions |
List of all members

stan::common::dump\_factory Class Reference

`#include <context_factory.hpp>`

Inheritance diagram for stan::common::dump\_factory:

|  |  |
| --- | --- |
| Public Member Functions | |
| stan::io::dump | operator() (const std::string source) |
|  | |
| Public Member Functions inherited from stan::common::var\_context\_factory< stan::io::dump > | |
|  | var\_context\_factory () |
|  | |

|  |  |
| --- | --- |
| Additional Inherited Members | |
| Public Types inherited from stan::common::var\_context\_factory< stan::io::dump > | |
| typedef stan::io::dump | var\_context\_t |
|  | |

## Detailed Description

Definition at line 19 of file context\_factory.hpp.

## Member Function Documentation

|  |  |  |  |  |  |
| --- | --- | --- | --- | --- | --- |
| stan::io::dump stan::common::dump\_factory::operator() | ( | const std::string | *source* | ) |  |

inlinevirtual

Implements stan::common::var\_context\_factory< stan::io::dump >.

Definition at line 21 of file context\_factory.hpp.

---

The documentation for this class was generated from the following file:

- src/stan/common/context\_factory.hpp


---

[ Stan Home Page ]

*© 2011–2014,
Stan Development Team.*
